# Global research trends in bacteriophage and gut microbiota: a bibliometric and visual analysis from 2012 to 2025

**DOI:** 10.3389/fmicb.2025.1738456

**Published:** 2026-01-16

**Authors:** Hui-Fang Kuang, Xiong-Yilang Jiang, Song-Yan Tie, Kun Lian, Mu-Yi Hao, Hang Xu, Xiao Huang, Yi Yang, Qian Guo, Jie Li, Ling-Li Chen

**Affiliations:** 1 School of Integrative Traditional Chinese and Western medicine, Hunan University of Chinese Medicine, Changsha, China; 2 Department of Joint and Trauma Orthopaedics, Xiangtan Chinese Medicine Hospital, Xiangtan, China; 3 College of Traditional Chinese Medicine, Hunan University of Chinese Medicine, Changsha, China; 4 Medical School of Hunan University of Chinese Medicine, Changsha, China

**Keywords:** bacteriophage, gut microbiota, bibliometric analysis, CiteSpace, VOSviewer

## Abstract

**Background:**

The gut microbiota constitutes a complex microbial ecosystem that plays a fundamental role in host metabolism and immune homeostasis. As the most abundant viral entities in the gut, bacteriophages are increasingly recognized as key modulators of microbial community structure and function. Nevertheless, the global research landscape and thematic evolution of bacteriophage–gut microbiota studies have not been systematically evaluated.

**Methods:**

Publications related to bacteriophages and the gut microbiota published between 2012 and 2025 were retrieved from the Web of Science Core Collection and Scopus databases. Bibliometric and visual analyses were conducted using CiteSpace, VOSviewer, and Scimago to examine publication trends, countries/regions, institutions, authors, journals, references, and research hotspots.

**Results:**

A total of 687 articles and reviews were included. The annual number of publications increased steadily, with accelerated growth after 2018 and a peak in 2023. China ranked first in publication output, while the United States demonstrated strong centrality in global collaboration networks. The University of California, San Diego and the University of Copenhagen were identified as leading institutions. Highly productive authors included Colin Hill, Bernd Schnabl, Zhang Yue, Li Shenghui, and Ross R. Pau. *Frontiers in Microbiology* and *Nature* are the most influential journals in this field. Keyword analyses revealed major research hotspots, including viral metagenomics, antimicrobial resistance, phage–microbiota–immune interactions, and the transition from phage therapy toward microecological and immunomodulatory interventions.

**Conclusion:**

Research on bacteriophage–gut microbiota interactions has shifted from descriptive profiling to mechanistic and translational studies, driven by advances in viral metagenomics and phage culturomics. Increasing attention has been directed toward disease-associated phage–microbiota interactions, particularly in inflammatory bowel disease, as well as the development of precision interventions such as phage therapy and engineered phages. This bibliometric analysis provides a comprehensive overview of global research trends and highlights emerging directions for future microbiome research.

## 1 Introduction

The gut microbiota, comprising bacteria, viruses, fungi, and archaea, constitutes a highly complex and dynamic microecosystem within the human body. This community plays a crucial role in health and disease prevention by regulating host metabolism and immune homeostasis (Guerin and Hill, 2020). With the rapid advancement of virology and metagenomics technologies, research on intestinal phages has emerged as a frontier in microbiology. Phages represent the most abundant viral population in the human gut, accounting for over 97% of the total gut virome (Gregory et al., 2020). Phages indirectly influence host immunity and disease progression by regulating bacterial abundance, metabolic functions, and horizontal gene transfer (Shkoporov et al., 2019; Rahimzadeh et al., 2021; Du et al., 2023). Unlike the non-selective killing of broad-spectrum antibiotics, phages exhibit high host specificity, enabling precise regulation without disrupting microbial balance (Hu et al., 2021). Phages maintain microbial stability through a lytic-lysogenic cycle: they coexist with hosts in a lysogenic state, they activate upon infection, lyse to release progeny, and reshape the microbiota (Chee et al., 2023). Research indicates phage colonization begins early in life, and is established through maternal transmission and environmental exposure, synchronizing with microbial diversity development (Rollie et al., 2020; Mahmud et al., 2024). Dietary patterns, antibiotic interventions, and inflammatory environments significantly influence phage-bacterial interaction networks, and their dysregulation correlates closely with diabetes, obesity, and cardiovascular diseases (Diard et al., 2017; Zou et al., 2022; Govender and Ghai, 2025).

As an emerging microbiome intervention strategy, phage therapy, demonstrates unique potential in both infectious and non-infectious diseases. Their host-specific lytic action enables precise elimination of pathogenic bacteria while avoiding antibiotic-induced dysbiosis and resistance (Fu et al., 2017). However, the phage–host–microbiome interaction mechanisms remain unclear, with some phages capable of activating the nuclear factor kappa-light-chain-enhancer of activated B cells (NF-κB) pathway or triggering systemic inflammation (Champagne-Jorgensen et al., 2023; Le et al., 2025). Furthermore, the CRISPR-Cas system’s interplay between phages and bacteria adds therapeutic complexity, prompting researchers to explore multivalent phage cocktails and engineered phages. Nevertheless, high costs and safety assessments remain significant bottlenecks (Naghizadeh et al., 2019). An estimated 80–90% of gut phages remain unannotated, with unclear host ranges and mechanisms governing their lytic-lysogenic cycle switching (Mahmud et al., 2024). Lytic phages may enhance bacterial virulence through horizontal gene transfer, however, systematic analysis of phage-microbiome-metabolite ecological networks remains insufficient. An example is the mechanism linking the microbial metabolite trimethylamine N-oxide to cardiovascular disease risk (Cazares et al., 2020).

Regarding clinical translation, phage therapy remains constrained by standardization and safety concerns. On one hand, phage preparations must comply with stringent sterility and genetic safety standards, yet highly effective lytic strains remain elusive for certain pathogens such as *Clostridium difficile* (Hargreaves and Clokie, 2014). On the other hand, clinical evidence primarily stems from case studies, lacking large-scale randomized controlled trials. Long-term efficacy for non-infectious diseases also necessitates monitoring microbial dynamics through metagenomics and metabolomics (Hsu et al., 2019). As a vital tool for analyzing research trends, scientometrics can identify research hotspots and frontiers by systematically analyzing authors, institutions, keywords, and co-citations (Diem and Wolter, 2013; Peng et al., 2023). Given phages’ potential value in combating antimicrobial resistance and regulating the microbiome, this study conducted a bibliometric analysis of phage and gut microbiota research from 2012 to 2025 using the Web of Science Core Collection (WOSCC) and Scopus databases, employing visualization tools such as CiteSpace and VOSviewer. The starting year of 2012 was selected because studies in this field were relatively limited and fragmented prior to this period, whereas advances in high-throughput sequencing and metagenomic technologies after 2012 enabled more systematic and reproducible research. The aim is to reveal the global research landscape and development trends, providing theoretical support for the precise regulation of the gut microbiome by phages and their clinical applications.

## 2 Materials and methods

### 2.1 Data sources and search strategy

**FIGURE 1:**
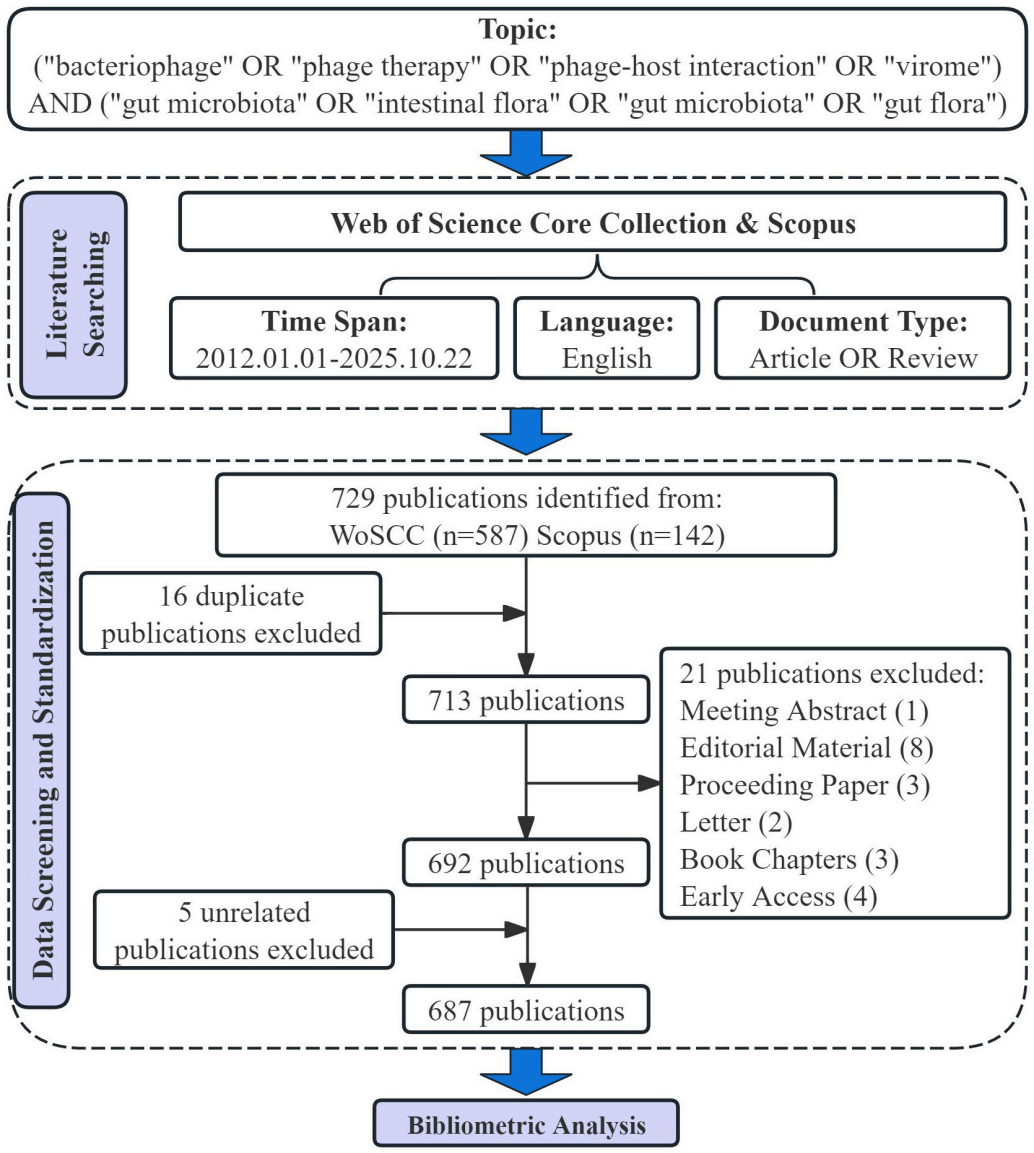
Flowchart of literature selection based on the PRISMA guidelines.

This study selected the Web of Science Core Collection (WOSCC) and Scopus as data sources, which cover high-quality scientific literature worldwide and are widely regarded as preferred tools for bibliometric analysis (Ding and Yang, 2020). An advanced search strategy was employed using the query: TS = (“bacteriophage” OR “phage therapy” OR “phage-host interaction” OR “virome”) AND (“gut microbiota” OR “intestinal flora” OR “gut microbiota” OR “gut flora”). The publication date range was restricted to January 1, 2012, to October 22, 2025, and only articles and review publications were included. The first author conducted the search and saved the data. The second and third authors independently screened the literature by reviewing titles and abstracts based on the inclusion/exclusion criteria, with disagreements resolved through consultation with the corresponding author. The exported metadata included titles, authors, keywords, citations, journals, institutions, and references, saved in plain text format (Chang et al., 2023). The study selection process was conducted in accordance with the PRISMA guidelines, and the inclusion and exclusion criteria are summarized in the PRISMA flowchart (Figure 1). In brief, records retrieved using predefined keywords were screened for relevance to bacteriophage regulation of the gut microbiome. Finally, CiteSpace, VOSviewer, and other bibliometric tools were used for multidimensional analysis and visualization of research outcomes. Since all data were obtained from public databases without involving human subjects or private information, ethics committee approval was not required.

### 2.2 Data analysis and visualization

The data analysis and visualization tools included CiteSpace (version 6.1.R6), VOSviewer (version 1.6.18), Scimago Graphica, and Microsoft Excel. Microsoft Excel was employed to calculate annual publication volumes by country/region and to generate line charts. CiteSpace and VOSviewer were used to visualize: (1) countries/regions and institutions; (2) authors and collaborative networks; (3) journal and reference co-citations; and (4) keyword co-occurrence clusters and bursts.

The visualization networks represent topological structures consisting of nodes and links. In these visualizations, node size corresponds to publication output, while link thickness indicates collaboration strength (Feng et al., 2023). VOSviewer provides three visualization modes: (1) network, (2) overlay, and (3) density visualizations, which collectively reveal cluster structures and identify pivotal nodes (Othman et al., 2022). Keyword co-occurrence analysis identified research hotspots within this field. In these visualizations, node size and font weight represent keyword frequency, whereas centrality values indicate conceptual importance. Nodes with centrality values ≥ 0.1 were classified as pivotal nodes. High-frequency, high-centrality keywords indicated established research priorities within the field. Keyword cluster analysis identified primary research directions. Lower cluster numbers corresponded to larger thematic groups. Cluster validity was assessed using Silhouette (S) and Modularity (Q) scores: S ≥ 0.5 indicated acceptable clustering; S ≥ 0.7 suggested strong reliability; and Q ≥ 0.3 denoted statistically significant cluster structures (Sabe et al., 2022; Isola et al., 2025). Temporal keyword emergence mapping identified evolving research frontiers. Emergence strength quantified intensity of sudden increases in keyword usage frequency, with higher values indicating more pronounced shifts. Journal Impact Factors were obtained from the Journal Citation Reports (2025 edition) in Web of Science.

To strengthen analysis validity, we normalized synonyms (e.g., “bacteriophage”/“bacteriophages,” “phage”/“bacteriophage,” “intestinal”/“gut”). For VOSviewer visualizations, we set display thresholds based on network density, including only items with connections. All other parameters used default settings. CiteSpace parameters included: analysis period (January 2012—October 2025) with yearly time slices. Pruning methods were “pathfinder” and “pruning sliced networks,” with all other parameters default.

## 3 Results

### 3.1 Literature search results

Our search retrieved 729 records, with 687 articles and reviews meeting the inclusion criteria after screening.

### 3.2 Analysis of publications and citations

**FIGURE 2:**
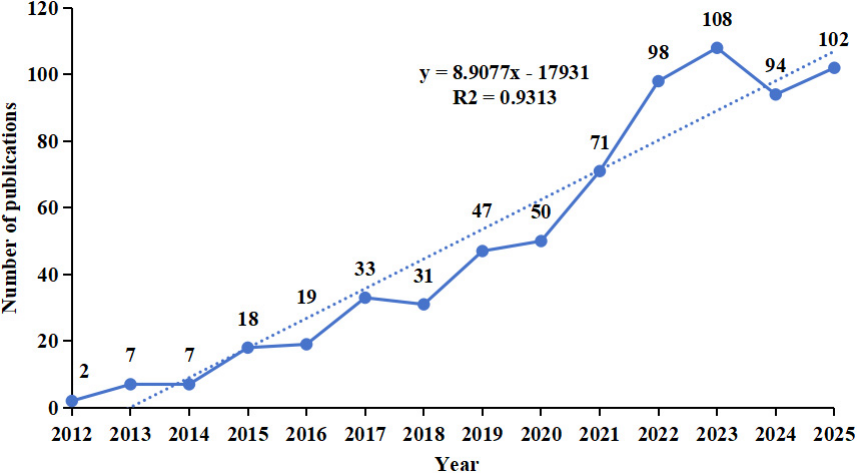
Annual number of publications in bacteriophage and gut microbiota research from 2012 to 2025. The dotted line represents the linear regression trend based on annual publication data from years with sufficient output for reliable fitting.

Publication metrics served as key indicators of research progress and field development. We used Microsoft Excel to analyze the annual number of publications related to phage and gut microbiota research (Figure 2). Annual publications showed consistent growth from 2012 to 2025. Publications peaked in 2023 (*n* = 108 articles). The recent upward trend suggests growing academic interest and field maturation.

### 3.3 Analysis of countries/regions

Sixty-three countries/regions contributed publications in this field, while Table 1 presents the top five most productive countries/regions. China produced the most publications (*n* = 174; 25.3%), followed by the United States (*n* = 156) and Italy (*n* = 36). These countries demonstrated both high productivity and central collaborative positions. Total Link Strength (TLS) analysis revealed the United States, United Kingdom, Germany, and China as key nodes in the collaboration network.

**TABLE 1:** Top five countries in publications on phage and gut microbiota.

Rank	Country	Publications	Total citations	Average citations	TLS	Centrality
1	China	174	4,597	26.42	35	0.15
2	The United States	156	12,978	83.19	124	0.52
3	Italy	36	2,842	78.94	29	0.09
4	Germany	34	1,873	55.09	45	0.13
5	The United Kingdom	33	3,122	94.61	51	0.19

**FIGURE 3:**
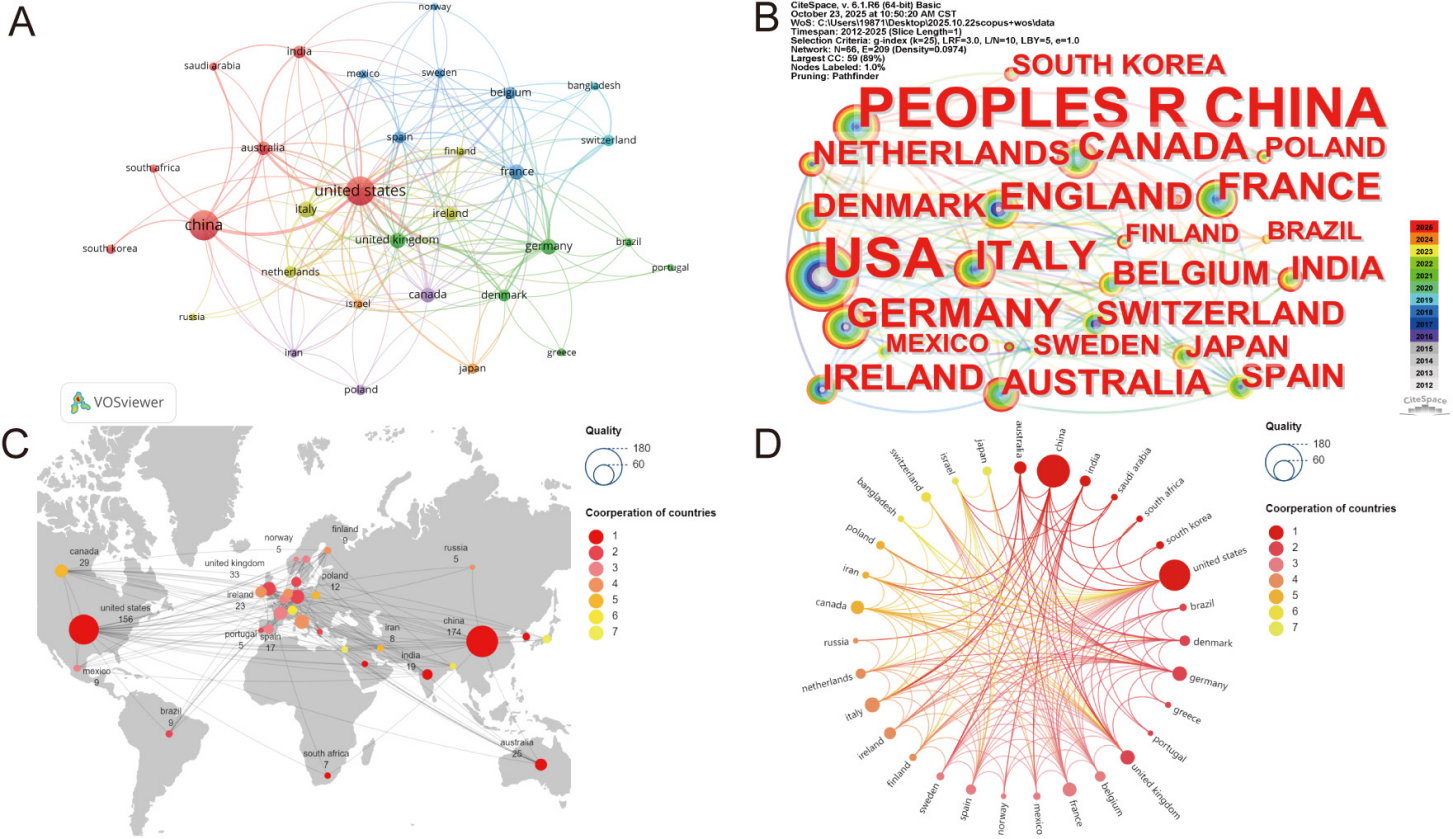
Visualization and analysis of the international collaboration networks in bacteriophage and gut microbiota research. **(A)** Cooperation clustering map of countries. **(B)** Cooperation map of countries by citations. **(C,D)** Country collaboration map by Scimago Graphica.

VOSviewer visualizations confirmed these patterns (Figures 3A–D). Thirty-one countries/regions published ≥ 5 papers each. The United States received the most citations, followed by China and the United Kingdom. The most frequent collaboration occurred between the United States and China, driving international knowledge exchange. This pattern shows how core nations disproportionately influence field advancement. However, research output remains unevenly distributed, with most publications originating from few countries. While promoting stable networks, this structure highlights the need for broader global participation. Such expansion would enhance resource sharing while reducing geographic bias and research duplication.

### 3.4 Analysis of institutions

**FIGURE 4:**
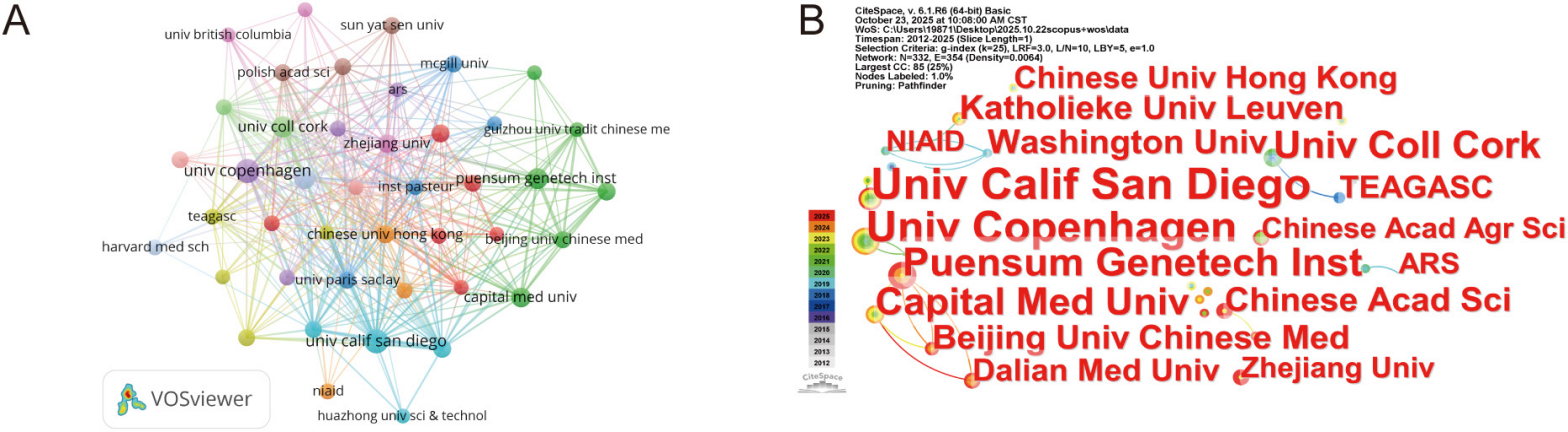
Visualization and analysis of the institutions in bacteriophage and gut microbiota research. **(A)** Cooperation map of 43 institutions with the number of publications no less than five times. **(B)** Centrality cooperation map of institutions.

A total of 1,081 institutions published articles in the field of phage regulation of the gut microbiota, with the top five institutions listed in Table 2. The University of California, San Diego and the University of Copenhagen produced the highest number of publications (17 each). VOSviewer analysis revealed 43 institutions with five or more publications. As shown in Figure 4A, different colors represent distinct collaboration clusters, with most clusters exhibiting intra-national collaboration patterns. Visual knowledge maps of research institutions generated using Citespace (Figure 4B) reveal that publications in phage-mediated gut microbiota regulation predominantly originate from a small number of institutions, such as the University of California, San Diego and the University of Copenhagen. Overall, achievements in this field are primarily driven by comprehensive public research universities with medical programs and internationally renowned institutions. Leveraging their resource platforms and academic strengths, these institutions play a central role in advancing higher-quality research and enhancing its impact.

**TABLE 2:** Top five institutions in publications on phage and gut microbiota.

Rank	Institution	Publications	Citations	Average citations/publication
1	University of California, San Diego	17	2,837	166.88
2	University of Copenhagen	17	901	53.00
3	University of Washington	13	2,345	180.38
4	University College Cork	13	899	69.15
5	Puensum Genetech Institute	12	71	5.92

### 3.5 Analysis of authors

**FIGURE 5:**
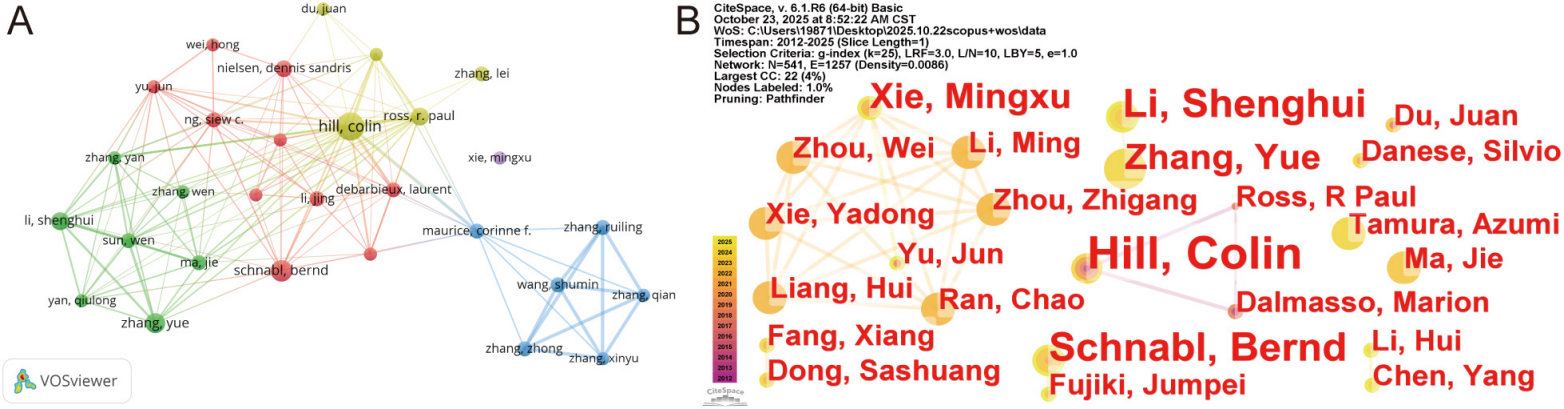
Visualization and analysis of the authors in bacteriophage and gut microbiota research. **(A)** Cooperation map of 29 authors with the number of publications no less than five times. **(B)** Centrality cooperation map of authors.

The included literature involved 3,842 authors, among whom 29 had published ≥ 5 papers (Figure 5). The co-occurrence network of authors generated by CiteSpace comprised 541 nodes and 1,257 connections, with a density of 0.0086. This low density indicates limited collaboration among authors, suggesting that a comprehensive large-scale collaborative network has yet to form. Five collaborative teams emerged, centered around Hill Colin, Schnabl, Bernd, Li, Shenghui, Xie, Mingxu, and Wang, Shumin. The most prolific author was Hill, Colin (15 papers), followed by Schnabl, Bernd (10 papers), Zhang, Yue (9 papers), and Li, Shenghui and Ross, R. Pau (both 8 papers) (Table 3). Author collaboration networks aim to reveal the most active and prolific authors and co-authors, visualize collaboration intensity, identify major collaborative teams and potential research partners within the field, and facilitate the establishment of closer collaborative networks (Xia et al., 2022). Figure 5 and Table 3 indicate that while numerous authors conduct research in this field, the overall collaboration structure remains fragmented, with relatively weak collaborative ties. This suggests a need to further strengthen cooperation and exchange between research teams, share clinical experience and research findings, and promote the development of high-quality research output in this field.

**TABLE 3:** Top five authors in phage and gut microbiota field.

Rank	Author	Documents	Citations	Countries/regions	Average citations/publication
1	Hill, Colin	15	896	Ireland	59.73
2	Schnabl, Bernd	10	629	The United States	62.90
3	Zhang, Yue	9	54	China	6.00
4	Li, Shenghui	8	45	China	5.63
5	Ross, R. Pau	8	660	Ireland	82.50

### 3.6 Analysis of references

**FIGURE 6:**
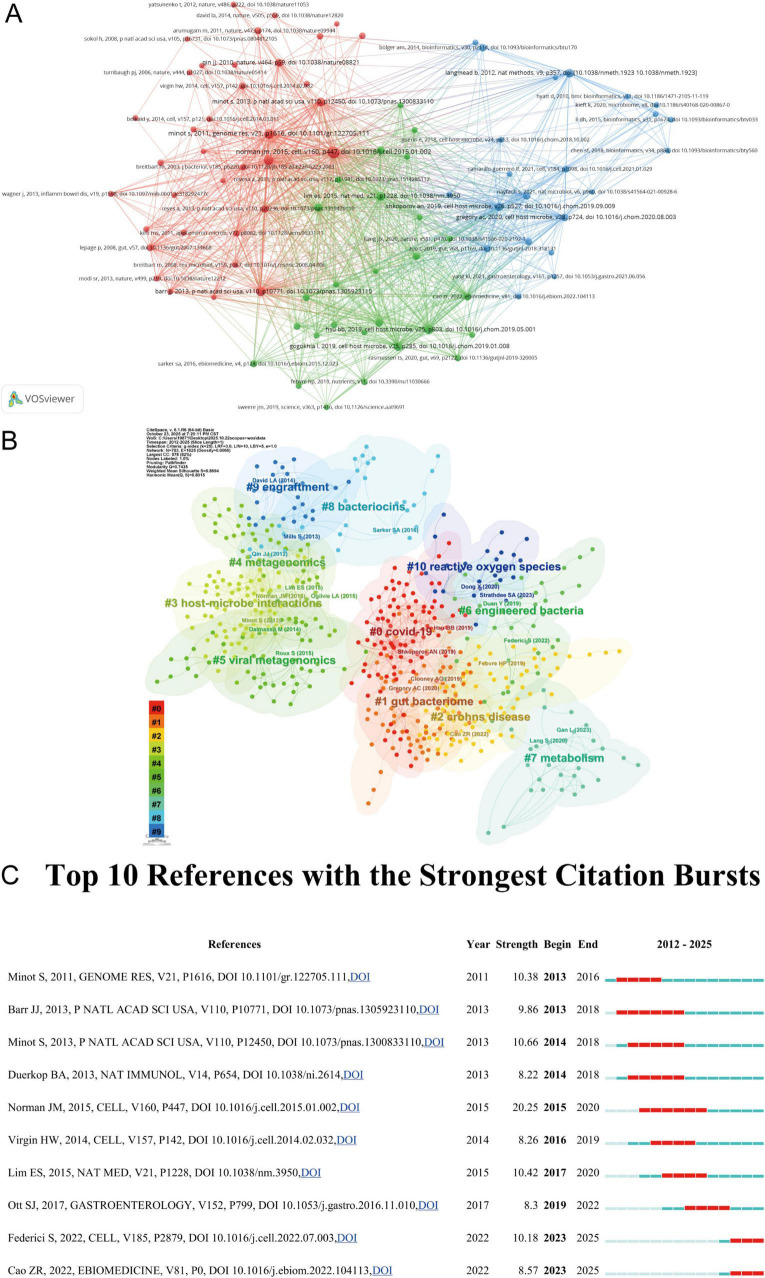
Visualization and analysis of the references in bacteriophage and gut microbiota research. **(A)** Distribution of 133 references with a frequency of no less than 20 times. **(B)** References co-citation clustering network. **(C)** Top 10 references with the strongest citation bursts.

A total of 687 articles cited 35,689 references (Table 4). The paper by Jason M Norman in 2015 received the highest number of citations (140), indicating its exceptional research value and influence in the field of phage regulation of the gut microbiota. Using VOSviewer for co-citation analysis, we set the minimum number of co-cited references to 20. This identified 133 articles meeting this threshold, forming three major clusters (Figure 6A). CiteSpace performed reference clustering analysis, identifying 11 major subtopics relevant to phage regulation of the gut microbiome. The Modularity Q-value was 0.7435 (> 0.3) and the Silhouette S-value was 0.8694 (> 0.7), indicating that the co-citation clusters were reliable and significant, with stable and dependable clustering results (Figure 6B). Citation emergence analysis (Figure 6C) revealed that Jason M Norman’s 2015 paper “Disease-specific alterations in the enteric virome in inflammatory bowel disease” exhibited the highest emergence intensity (20.25), reflecting its significance within the field of phage-mediated gut microbiota regulation. Through multi-cohort metagenomic sequencing with cross-regional validation, combined with virus-like particle (VLP) enrichment and 16S rRNA analysis, this study systematically revealed the dynamic changes in the viral microbiome associated with inflammatory bowel disease (IBD) and its impact on host pathophysiology. The study not only provides crucial evidence for assessing viral risks in inflammation and dysbiosis but also lays the groundwork for developing viral diagnostic tools and phage-targeted therapies, advancing research into the interaction mechanisms between the gut virusome and microbiome.

**TABLE 4:** Top 10 references in phage and gut microbiota field.

Rank	Author	Co-citations	Year	Journals	DOI
1	Jason M. Norman	140	2015	Cell	10.1016/j.cell.2015.01.002
2	Alejandro Reyes	92	2010	Nature	10.1038/nature09199
3	Samuel Minot	90	2011	Genome Research	10.1101/gr.122705.111
4	Jeremy J. Barr	80	2013	Proceedings of the National Academy of Sciences of the United States of America	10.1073/pnas.1305923110
5	Bryan B. Hsu	72	2019	Cell Host Microbe	10.1016/j.chom.2019.05.001
6	Andrey N. Shkoporov	69	2019	Cell Host Microbe	10.1016/j.chom.2019.09.009
7	Efrem S. Lim	68	2015	Nature Medicine	10.1038/nm.3950
8	Ben Langmead	68	2012	Nat Methods	10.1038/nmeth.1923 10.1038/nmeth.1923
9	Lasha Gogokhia	66	2019	Cell Host Microbe	10.1016/j.chom.2019.01.008
10	Samuel Minot	63	2013	Proceedings of the National Academy of Sciences of the United States of America	10.1073/pnas.1300833110

### 3.7 Analysis of journals

**FIGURE 7:**
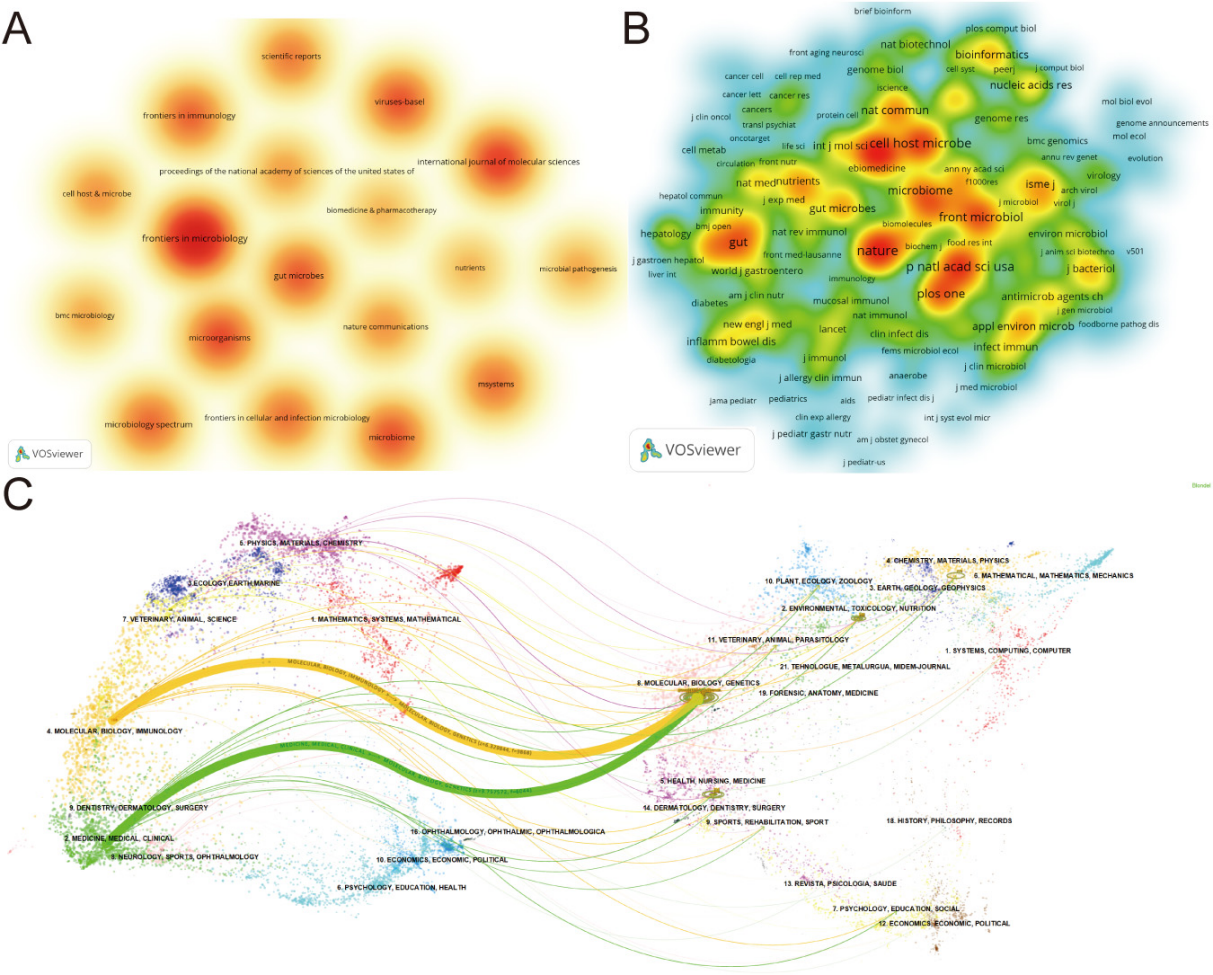
Visualization and analysis of the top journals in bacteriophage and gut microbiota research. **(A,B)** Density visualization of journals and co-cited journals in bacteriophage and gut microbiota field. **(C)** Dual-map of journals on bacteriophage and gut microbiota research.

The 687 articles in this field were published across 273 journals, with 20 journals publishing ≥ 5 articles. Frontiers in Microbiology published the most articles (*n* = 36), followed by the International Journal of Molecular Sciences (*n* = 20) and Microorganisms (*n* = 17). Figures 7A,B and Table 5 present the top 10 journals by publication volume and citation impact. Dual-map overlay analysis can be used to examine the distribution characteristics of disciplines and journals and reveal connections between them. The left side represents the disciplinary distribution of citing journals, while the right side shows the disciplinary distribution of cited journals. Paths of different colors indicate citation relationships between them (Ma et al., 2021; Jiang et al., 2025), with two paths representing the disciplinary transition links between citing and cited journals. Dual-map overlay analysis indicates that citing literature related to phage regulation of gut microbiota predominantly appears in journals of Molecular/Biology/Immunology, Medicine/Medical/Clinical, Dentistry/Dermatology/Surgery, and Neurology/Sports/Ophthalmology, while cited literature primarily originates from Molecular/Biology/Genetics journals. This pattern suggests phage-gut microbiota research emerged from fundamental life science advances. Due to its immense application potential, it rapidly attracted participation from various clinical medical disciplines. This demonstrates that the field’s development relies on deep integration between disciplines such as molecular biology, genetics, microbiology, and bioinformatics with clinical medicine and various clinical specialties, ultimately forming a highly interdisciplinary research paradigm. Analysis reveals that journals in this field exhibit high impact factors and quality standards, ensuring the rigor and academic integrity of published research.

**TABLE 5:** Top 10 journals in number of publications and citations in phage and gut microbiota field.

Rank	Publication journal	Documents	Citations	IF*	Cited journal	Co-citations	IF*
1	Frontiers in Microbiology	36	1,188	4.5	Nature	1,669	48.5
2	International Journal of Molecular Sciences	20	451	4.9	Cell Host and Microbe	1,345	18.7
3	Microorganisms	17	445	4.2	Proceedings of the National Academy of Sciences of the United States of America	1,331	9.1
4	Microbiome	16	1,680	12.7	PLoS One	1,175	2.6
5	Viruses-Basel	16	458	3.5	Science	1,116	45.8
6	Gut Microbes	15	442	11	Frontiers in Microbiology	1,090	4.5
7	Frontiers in Immunology	14	251	5.9	Cell	999	42.5
8	Microbiology Spectrum	13	136	3.8	Gut	959	25.8
9	Frontiers in Cellular and Infection Microbiology	12	189	4.8	Scientific Reports	840	3.9
10	mSystems	12	438	4.6	Nature Communications	779	15.7

IF*, impact factor.

### 3.8 Analysis of keywords in phage and gut microbiota research

**FIGURE 8:**
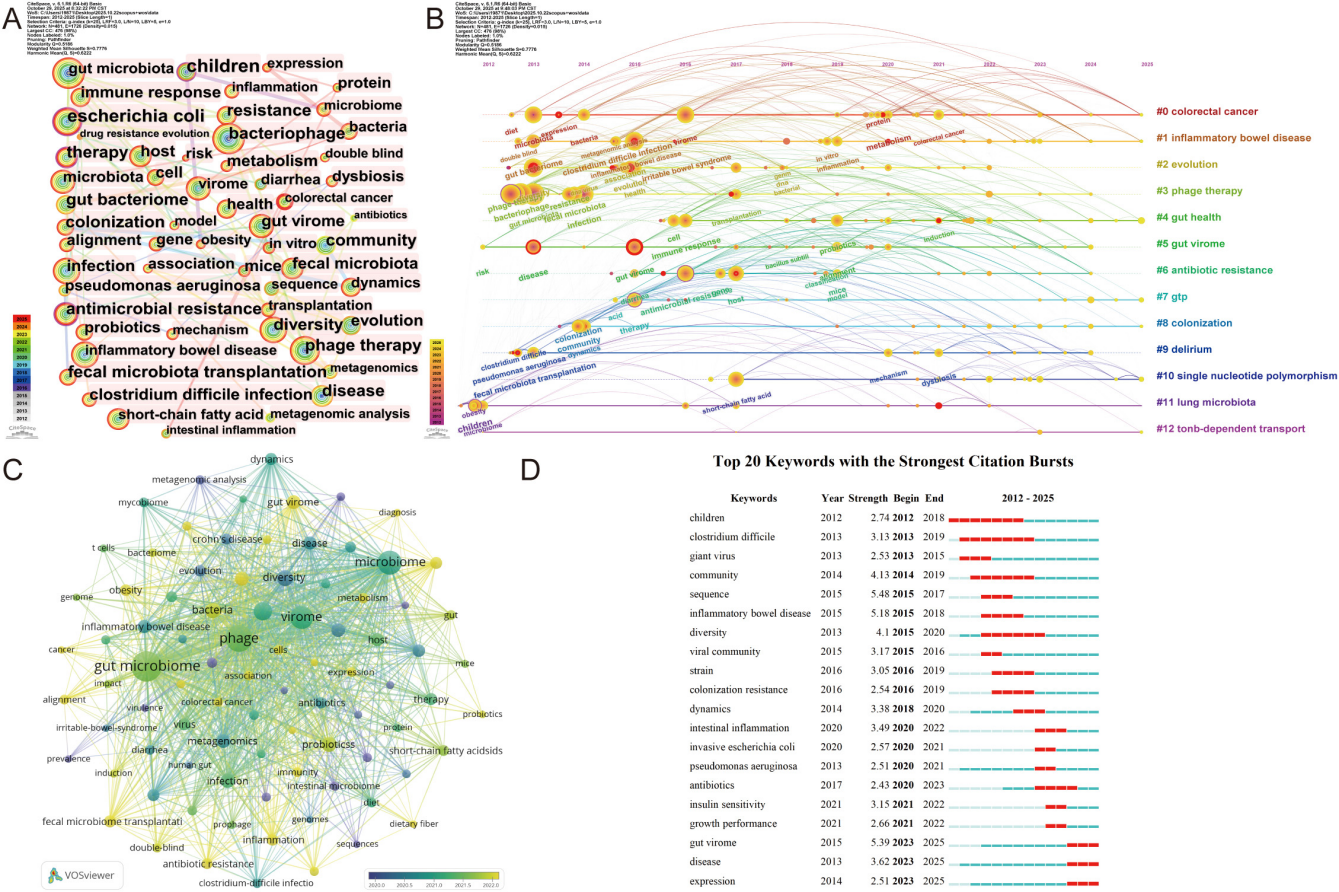
Visualization and analysis of the keywords in bacteriophage and gut microbiota research. **(A)** Co-occurrence of keywords. **(B)** Timeline view of keywords cluster. **(C)** Distribution of 87 keywords with an average publication of no less than 10 times. **(D)** Top 20 keywords with the strongest citation bursts in literature related to bacteriophage and gut microbiota research.

Keywords encapsulate a study’s core concepts and thematic focus. Systematic keyword analysis identifies research themes through high-frequency terms, revealing field developments and emerging directions (Fu et al., 2023). Keyword co-occurrence mapping visually classifies research themes, highlighting current foci and trends. The keyword co-occurrence map is shown in Figure 8A. High-frequency keywords comprised: phage therapy, gut virome, inflammatory bowel disease, diversity, and bacterial infection. The top 10 keywords by frequency are listed in Table 6. Key nodes in this study include Children (0.14), Therapy (0.11), Immune response (0.10), Antimicrobial resistance (0.10), Community (0.10), and Colonization (0.10). The keyword clustering network diagram is shown in Figure 8B. Results indicate a literature clustering S-value of 0.7776 and a clustering Q-value of 0.5186, confirming the clustering as statistically significant and reliable. The 13 clusters reflected major research trajectories and temporal evolution. Further summarizing each cluster’s research focus, the 13 clusters were consolidated into four major categories, as shown in Table 7. 1. Virome and metagenomic innovations: This category represents technology-driven frontier exploration, focusing on revealing the diversity, community structure, and functions of the gut virome through novel techniques such as viral metagenomics and high-throughput sequencing. It aims to unravel the “viral dark matter” and provide a methodological foundation for understanding phage-host interactions. 2. Resistance evolution and host-microbe interactions: This category focuses on molecular and ecological mechanisms, emphasizing bacterial adaptive evolution under environmental pressures like antibiotics and host immunity. Examples include the “arms race” between CRISPR-Cas systems and anti-CRISPR proteins, and the role of microbial metabolites such as short-chain fatty acids (SCFAs) in regulating host signaling pathways and outcomes of bacterial infections. 3. From phage therapy to microecological intervention: This category signifies a paradigm shift from single-pathogen eradication to holistic microecological regulation. Core strategies include targeted bacterial killing using natural or engineered phages, alongside microecological interventions like fecal virus community transplantation to restore gut microbial balance for disease treatment. 4. Immunomodulation through microbiome engineering: This category focuses on host-level mechanisms and applications, delving into how dysbiosis triggers chronic inflammatory diseases by disrupting the intestinal barrier, altering immunomodulatory molecules like toll-like receptors, and affecting SCFAs. It also evaluates intervention strategies such as Fecal Microbiota Transplantation (FMT) in improving disease outcomes by restoring immune homeostasis. Figure 8D presents keyword salience analysis. “Sequence” showed highest salience (5.48), with temporal analysis revealing two phases: Phase I (2012–2019) emphasized: pediatric studies, *C. difficile*, viral ecology, and colonization resistance. Key themes included gut disease and pathogen studies, community structure and diversity analysis, viral ecology exploration, and colonization and resistance mechanisms. Phase II (2020-present) shifted toward: inflammatory mechanisms, *E. coli/P. aeruginosa* pathogenesis, and virome functional dynamics. Building upon studies of community ecology and resistance mechanisms, this phase expands into inflammation and immune regulation, metabolic health and host interactions, and functional remodeling of the gut virome. This shift reflects an evolution in research focus from structural characteristics toward functional mechanisms and clinical translation. Keywords such as Gut virome, Disease, and Expression have emerged as prominent current research hotspots.

**TABLE 6:** Top 10 keywords by frequency and centrality in phage and gut microbiota field.

Rank	Keywords	Frequency	Keywords	Centrality
1	Gut microbiota	396	Children	0.14
2	Bacteriophage	202	Therapy	0.11
3	Phage therapy	107	Immune response	0.10
4	Virome	104	Antimicrobial resistance	0.10
5	Gut virome	70	Community	0.10
6	Diversity	69	Colonization	0.10
7	Microbiota	59	Gut virome	0.09
8	Infection	58	Gut bacteriome	0.09
9	Bacteria	56	Infection	0.08
10	Inflammatory bowel disease	55	Disease	0.08

**TABLE 7:** Main research directions and representative keywords on phage and gut microbiota.

No.	Research direction	Cluster ID	Representative keywords (LLR)
1	Virome and metagenomic technological innovations	#2, #5, #6	Viral metagenomics, viral diversity, gut virome, viral function, metagenome, sequence, viromics, phage gene sequencing.
2	Resistance evolution and microbiota-immune interactions	#2, #6, #7, #12	Antibiotic resistance, evolution, wild avian, anaerobically cultivated human intestinal microflora (achim), gtp, lipid metabolism, bacterial infections, tonb-dependent transport, plague, colicin.
3	From phage therapy to microecological intervention	#3, #4, #8	Phage therapy, bacteriophage, transplantation, ulcerative coliti, gut health, lytic cycle, bacteriophage crispr engineering, colonization, fecal virome transplantation, fecal virome.
4	Immunomodulation and microecological intervention	#0, #1, #9, #10, #11	Inflammatory bowel disease, colorectal cancer, dysbiosis, immuno-regulation, intestinal permeability, short-chain fatty acids, fecal microbiota transplantation, lung microbiota, toll-like receptors, gut bacteriome.

## 4 Discussion

### 4.1 Overall distribution

Since the early 20th century, epidemiological transitions have shifted global mortality patterns from infectious to non-communicable diseases (GBD 2019 Diseases and Injuries Collaborators, 2020). Consequently, host-microbiota interactions have emerged as a key research focus in biomedicine. Mounting evidence links gut dysbiosis to chronic diseases (cardiovascular, IBD, neurological, and allergic disorders), representing a global health challenge (Kayama et al., 2020). However, phage roles in microbial networks and host health regulation remain underexplored (Avellaneda-Franco et al., 2024).

Our analysis included 687 English publications (2012–2025). Publication volume increased from 2 papers in 2012 to 108 in 2023, showing a sustained upward trend with particularly notable growth during critical junctures like pandemic outbreaks. This indicates that as global public health events become more frequent, research on the association between phages and gut microbiota is gaining increasing attention from the global academic community. Its importance in fields such as health and disease prevention and control is continuously rising, suggesting that the practical demand for prevention and control drives the deepening of research. From a national perspective, China produced the highest number of publications, whereas the United States exhibited the strongest total link strength, indicating a more extensive international collaboration network. Notably, Western countries such as the United Kingdom, the United States, and Italy showed relatively higher average citations per publication, reflecting greater research impact. These patterns may be attributed to well-established research infrastructure and long-term investment in microbiome studies, earlier entry into the field allowing more time for citation accumulation, and higher international visibility resulting from English-language publications in high-impact or open-access journals (Zhou et al., 2020; Ryan et al., 2021). Institutions such as the University of California, San Diego and the University of Copenhagen have played a pivotal role in advancing global microbiome research through their outstanding contributions to understanding the mechanisms underlying gut microbiota dysbiosis, metabolic diseases, and phage interactions (Tanase et al., 2020; Zaky et al., 2021). However, geographic concentration and collaboration fragmentation remain challenges, requiring strengthened international cooperation. Future efforts should foster transnational scientific cooperation and multidisciplinary integration to establish a more systematic and collaborative global research network in this field. Collaboration networks revealed influential research teams led by Colin Hill, Bernd Schnabl, and R. Paul Ross. Colin Hill’s team spans diverse research domains, from phage structural biology to clinical microbiome interventions (King, 2024). His work pioneered the atomic-level structure of crAssvirus—the most abundant phage in the human gut—via cryo-electron microscopy, providing foundational theoretical insights into phage assembly and infection mechanisms (Shkoporov et al., 2018; Bayfield et al., 2023). His team pioneered standards for microbial therapeutics and postbiotics while translating microbiome research into clinical practice (O’Toole et al., 2017; Salminen et al., 2021; Mosca et al., 2022). Colin Hill’s research centers on the dynamic symbiotic relationship between phages and host bacteria. For instance, his research proposed the “phage cocktail therapy” strategy and demonstrated its ability to significantly reduce pathogenic bacterial abundance *in vitro* models (Buttimer et al., 2022; Cortés-Martín et al., 2025). Additionally, he introduced the concept of fecal viral transfer (FVT), proposing that phages play a crucial role in gut microbiota restoration following antibiotic interventions. This framework facilitates the development of non-virographic formulations and offers novel insights (Draper et al., 2020). Furthermore, the team elucidated the mechanisms linking phages to metabolic diseases and IBD, emphasizing synergistic strategies combining narrow-spectrum antibiotics with probiotics to counter the spread of resistance genes caused by antibiotic overuse (Murphy et al., 2013; Clooney et al., 2019; O’Connor et al., 2025). These studies not only shape the knowledge structure of this field but also advance the understanding of phage-gut microbiota-host interactions, providing a theoretical foundation for precision microbiome interventions and personalized therapies. Key findings appear predominantly in high-impact journals like Frontiers in Microbiology and International Journal of Molecular Sciences. This interdisciplinary research bridges microbiology, chemistry, and biomedicine, focusing on mechanistic insights and clinical translation (Mei et al., 2022; Zhou et al., 2023). Most journals feature high impact factors, with JCR rankings predominantly in Quartiles 1 and 2, reflecting the field’s overall high research quality and steadily increasing academic influence.

### 4.2 Research hotspots

Bibliometrics systematically analyzes publication data to reveal research trends and patterns (Li et al., 2024). Keyword analysis (co-occurrence, clustering, emergence) identifies research themes, while co-citation networks map intellectual foundations Thus, keyword analysis in phage-microbiota research helps forecast emerging directions (Chen, 2017; Wang J. H. et al., 2024). Our analysis identified four research hotspots: innovations in virology and metagenomics technologies, evolution of drug resistance and microbiota-immune interactions, progression from phage therapy to microbiome modulation, and immune regulation and microbiome interventions.

#### 4.2.1 Virome and metagenomic technological innovations

Phages modulate gut microbiota structure and function via lytic-lysogenic cycles (Molan et al., 2022). Viromics—the study of viral communities—has revolutionized gut viral diversity and functional characterization. However, viromics historically trailed microbiomics due to technical challenges: sample complexity, viral diversity, and unannotated sequences (“viral dark matter”). Advanced sequencing and bioinformatics now enable functional virome analyses beyond descriptive studies (Yu et al., 2024). Viral metagenomics tracks spatiotemporal dynamics, host interactions, and functions of gut phages (González and Elena, 2021; Santos-Medellin et al., 2021; Zuo et al., 2021; Muscatt et al., 2023). For instance, Bonilla-Rosso et al. (2020) utilized metagenomics to uncover the community structure and host associations of bee gut viruses, providing a model for studying complex viral ecosystems. Dion et al. (2020) demonstrated how phage genome mosaicism drives structural and community diversity, linking viral evolution to microbiome stability. Overreliance on fecal samples has limited understanding of mucosal-luminal virome dynamics. Whole-gut virome sequencing reveals ecosystem-scale viral diversity. Recent studies demonstrate significant differences between the mucosa-associated viral community and the fecal virome, including abundant crAss-like phages that are difficult to detect in fecal samples. Yan et al. (2023) combined multi-omics approaches to characterize viral activity and phage-bacteria interactions in IBD.

Recent research increasingly focuses on functional phage-microbiota interactions. Gut phages maintain physiological functions such as microbiota balance, fiber degradation, nutrient cycling, and gene transfer through complex interactions with the gut microbiota (Gilbert et al., 2020). Technological advances in high-throughput sequencing have not only accelerated the discovery of novel viruses but also revealed their distribution within human tissues, deepening our understanding of their biological roles (Foulongne, 2015). From a tool perspective, VIBRANT enables automated viral genome reconstruction and annotation through machine learning and protein similarity assessment, significantly enhancing the accuracy of functional predictions (Kieft et al., 2020). Integration with deep sequencing improves virus discovery while standardizing experimental design for reproducible metagenomics.

In summary, innovations in virology and metagenomics have expanded research from single fecal samples to multi-ecological niche systematic exploration, evolving from merely “seeing” viral communities to “deciphering” their functions, evolution, and host interactions. This enables systems biology approaches through multi-omics integration. These advances illuminate microbiome-health relationships and enable novel diagnostics/therapeutics.

#### 4.2.2 Resistance evolution and microbiota-immune interactions

Bacterial pathogenicity and antibiotic resistance evolution represent a critical global health challenge (de Kraker et al., 2011). Drug-resistant bacteria evolve via: horizontal gene transfer, mutation accumulation, and ecological competition. MDR strains (e.g., MRSA, MDR-*E. coli*) employ pili adhesion, biofilm formation, and TonB systems to enhance colonization and immune evasion (De Nisco et al., 2019; Schwartz et al., 2023). The TonB system mediates both pathogen virulence and phage-bacteria coevolution. It can serve as a gateway for phage invasion or limit infection, thereby driving co-evolution and continuous shifts in selective pressures. This phage-bacteria competition occurs in both laboratory and natural systems (e.g., avian hosts), demonstrating its ecological ubiquity. Anaerobically cultured human intestinal microbiota (ACHIM) enables precise study of phage integration, lysis, and resistance gene transfer (Fretheim et al., 2025). Bacterial phage defenses include: CRISPR-Cas, restriction-modification, and toxin systems (Spriewald et al., 2020). Phages counter with anti-CRISPR proteins, methylation, and recombination to maintain infectivity (Studier, 1975; Bikard and Marraffini, 2012; Bondy-Denomy et al., 2013; Shabbir et al., 2016). For instance, the T7 phage Ocr protein mimics host DNA-binding restriction enzymes to block cleavage and evade host restriction systems (Studier, 1975). Meanwhile, AcrF1 and AcrF2 in Pseudomonas phage DMS3v suppress the I-F CRISPR system, granting phages a dynamic advantage in the “attack-defense” cycle (Bondy-Denomy et al., 2013). This phage-bacteria coevolution involves complex ecological dynamics beyond simple attack-defense interactions. It constitutes a systemic process deeply intertwined with host nutrition, infection ecology, and resistance diffusion, forming a continuously dynamic equilibrium force within the microbiome.

Phage-microbiota coevolution interacts with host immune networks through multiple pathways. Host GTP-binding proteins play a central role in regulating antibacterial autophagy, inflammasome activation, and immune signaling, serving as key nodes linking phage infection to immune homeostasis (Shahsavari et al., 2022). Phage-mediated bacterial lysis releases cellular debris and lipid metabolism intermediates, participating in intestinal lipid metabolism and thereby influencing inflammatory responses and metabolic homeostasis (Cuomo et al., 2024). As the “training ground” for the immune system, gut microbiota dysbiosis has been implicated in numerous diseases, including IBD (Bai et al., 2022), cardiovascular disease (Piccioni et al., 2021), diabetes (Adeshirlarijaney and Gewirtz, 2020), neurodegenerative disorders (Ghyselinck et al., 2021; Geng et al., 2022), and psychiatric conditions such as anxiety and depression (Chen et al., 2020). Research indicates that phages alter gut-liver axis signaling and systemic immune responses by influencing microbial community structure and metabolite composition. For instance, modulating Akkermansia abundance reduces lipid peroxidation in non-alcoholic steatohepatitis (Jiang et al., 2024), while punicalagin improves colitis models by enriching beneficial bacteria (Liu et al., 2024). Yadan et al. demonstrated that DNA nanoparticle-modified *H. pylori*-specific phages not only achieve effective delivery to the gastrointestinal tract but also significantly restore colon length, reduce inflammation, and improve gut microbial diversity by reshaping the intestinal microenvironment in IBD. Compared to current clinical treatments, this approach effectively prevented colon tumor development in mouse models (Zhao et al., 2025). Phages show therapeutic potential beyond antibacterial applications, including immune-metabolic regulation. Through integrated multi-omics and ecological modeling, cross-scale evidence from wild avian to ACHIM ecosystems progressively reveals phages’ multifaceted roles in driving antibiotic resistance evolution, shaping microbial community functions, and modulating host immunity. Future integration of research on TonB system-mediated nutrient competition, GTP signaling networks, and lipid metabolism pathways will further elucidate the systemic regulatory mechanisms of phages in microbiota-immune interactions, providing novel theoretical support for the prevention and control of drug-resistant infections and microbiome-based therapies.

#### 4.2.3 From phage therapy to microecological intervention

Phage therapy constitutes a major breakthrough in addressing antibiotic resistance. Current research is transitioning from pathogen-specific approaches to precision microbiome modulation. Lytic phages effectively target resistant pathogens including *E. coli* and *C. difficile* in experimental models (Bolocan et al., 2016; Heuler et al., 2021; Shamsuzzaman et al., 2024). However, traditional phage therapy faces challenges including narrow host range, bacterial resistance, and immune clearance (Khambhati et al., 2023). Synthetic biology approaches enable engineered phages to combat antibiotic resistance through targeted mechanisms. CRISPR-Cas phage engineering can disrupt resistance genes (e.g., *bla_*NDM*–1_*, *mecA*) and reverse resistance (Wadan et al., 2025). Phages engineered with β-lactamase inhibitors or efflux blockers synergize with antibiotics (Sun et al., 2023; Cristinziano et al., 2024). Engineered phages deliver biofilm-degrading enzymes and virulence-modulating proteins (Fischetti, 2018; Le et al., 2024). Simultaneously, anti-CRISPR proteins like AcrIIC4 are employed to suppress host defenses, prolonging phage activity within the body (Torres-Boncompte et al., 2025). Emerging phage-probiotic systems precisely target pathogens while preserving microbiota balance (Shen et al., 2023). In IBD models, these systems remodel microbiota and reduce inflammation, revealing phages’ dual role as antimicrobials and ecological modulators. Furthermore, combination therapy involving phages with antibiotics or immune checkpoint inhibitors exhibits synergistic enhancement effects (Xiao et al., 2024; Démoulins et al., 2025). For instance, phages can enhance antitumor immune responses by promoting drug penetration or restoring T-cell function (Chen et al., 2023). The 2024 European Pharmacopoeia’s inaugural publication of quality standards for phage therapeutics (Fürst-Wilmes et al., 2025) signifies the field’s transition from experimental validation to standardized clinical translation. Despite challenges (immunogenicity, manufacturing), phage therapy now enables designed ecological interventions for precision medicine.

In tandem with phage therapy, microbiome intervention strategies represented by probiotics, prebiotics, FMT, and fecal virome transplantation (FVT) are establishing a therapeutic system jointly regulated by the “microbiota-phage-host” triad. Traditional FMT restores intestinal homeostasis by replenishing microbial diversity and functional redundancy, while FVT extends the concept of “microbial transplantation” by introducing the fecal virome as a key ecological regulatory layer. This approach accelerates microbiota reconstitution, promotes beneficial bacterial colonization, and reshapes host immune responses (Bornbusch et al., 2024; Yarahmadi et al., 2024; Yoo et al., 2025). In multiple models, such as antibiotic-disturbed cheetahs and metabolic syndrome mice, both FMT and FVT significantly restored gut homeostasis while improving energy metabolism and inflammation levels (Bornbusch et al., 2024; Yarahmadi et al., 2024). Probiotic interventions also demonstrate systemic regulatory potential, with specific strains improving metabolic disorders and neurobehavioral deficits by producing SCFAs and neurotransmitter precursors (Mushraf et al., 2024; Sharma et al., 2025). In cancer therapy, gut microbiota structure and function have been shown to directly influence immune checkpoint inhibitor efficacy (Nobels et al., 2025), while combined phage and microbiota interventions enhance treatment response by modulating the immune microenvironment. Notably, phages exert enduring ecological effects on gut colonization and virome dynamics, acting as “ecological amplifiers” post-FMT/FVT to restore microbial balance and stabilize immune-metabolic networks, thereby improving overall gut health. Collectively, research is progressively shifting from a “pathogen control” paradigm toward “ecological reshaping”; from phage therapy to microbiome restoration. Integrating engineered phages with multi-layered microbiome interventions holds promise for establishing novel therapeutic models centered on gut health, offering new directions for systematic precision treatment of complex diseases such as metabolic disorders, IBD, and cancer.

#### 4.2.4 Immunomodulation and microecological intervention

Host-microbiome interactions constitute a core mechanism maintaining physiological homeostasis and infection defense (Letizia et al., 2025). Gut microbiota regulate dendritic cell and Treg differentiation via SCFAs, mediating mucosal and systemic immunity (Sun et al., 2025). Intestinal barrier integrity maintains microbiota-immune balance during homeostasis (Mukhopadhya and Louis, 2025). Toll-like receptors (TLRs) mediate gut-liver crosstalk through Microbe-Associated Molecular Patterns (MAMPs) recognition, activating TLR4/MyD88/NF-κB signaling (Behzadi et al., 2021; Dong et al., 2024; Wang Z. et al., 2024). Metabolic dysfunction-associated steatotic liver disease progression correlates with dysbiosis, lipid accumulation, and insulin resistance. These factors can compromise the intestinal barrier and increase permeability, forming a chronic inflammatory pathway via the “gut-liver axis” (Benedé-Ubieto et al., 2024). Chronic dysbiosis disrupts tight junctions and hyperactivates TLRs, driving persistent inflammation that promotes IBD and colorectal cancer (Koleva et al., 2024; Wang J. et al., 2024; Zeng et al., 2025). SCFAs function as both energy sources and immune regulators via GPR41/43 receptors, maintaining metabolic-immune homeostasis (Mei et al., 2024). Crucially, the influence of intestinal immune signaling extends beyond the local mucosa to distant organs including the lungs, liver, and central nervous system. The gut-lung axis enables lung microbiota to modulate respiratory immunity through cross-talk with gut microbes (Özçam and Lynch, 2024). Meanwhile, the gut–brain axis transmits metabolic signals from the gut microbiota to the central nervous system via neuropathways such as chemosensory epithelial cells and the vagus nerve, thereby regulating mood, cognition, and gastrointestinal function (Ohara and Hsiao, 2025). Chronic inflammation, metabolic dysregulation, and suppression of immune surveillance induced by microbial products are recognized as key mechanisms by which gut microbiota promote carcinogenesis. Their pivotal role in malignant transformation within the hepatobiliary system has also been confirmed (Li et al., 2025). Thus, cross-organ immune–microbiome interactions not only reveal novel pathological patterns of systemic inflammation but also provide a theoretical foundation for microbiome-based interventions.

Phage-FMT integration marks a major advance in immunomodulatory microbiome research (Yadegar et al., 2024). Engineered phages enable targeted bacteriolysis and host range expansion via receptor protein editing, offering novel antimicrobial strategies (Latka et al., 2021). 7-deazaguanine modifications enhance phage evasion of bacterial defenses including CRISPR-Cas systems (Kot et al., 2020; Olsen et al., 2023). By prolonging their *in vivo* efficacy, these modifications drive alterations in their interaction patterns with the host immune system. FMT mechanisms now encompass virus-host immune co-regulation beyond bacterial transfer. It not only reconstructs microbial composition and restores microbiome homeostasis but may also enhance host defense through phage-mediated immune modulation (Piel et al., 2022; Rooney et al., 2023). Additionally, synergistic ecological strategies combining probiotics and phages can eliminate pathogens while preserving commensal communities, achieving dual effects of ecological balance and immune stability (Wang et al., 2021). Moreover, the integration of microbiome interventions with cell therapies represents an emerging trend. By enhancing immune cell metabolism and mucosal colonization capacity, this approach may establish a “dual-target immune-microbiome” regulatory system with potential value in systemic immune reconstruction (Peng et al., 2025). These studies underscore that future research must not only explore complex immune-microbiome interaction networks but also develop combination therapies integrating engineered phage, FMT, and immune modulation strategies for chronic inflammation, tumors, and immune disorders. These studies aim to advance translational applications from localized microbiome restoration to systemic immune remodeling.

### 4.3 Limitation

While this study offers valuable insights into phage-microbiome research trends, some limitations should be noted: First, using only WoSCC and Scopus databases may have excluded relevant studies from PubMed and other sources. This choice was made to ensure standardized citation metadata and compatibility with bibliometric analysis tools, while minimizing potential duplication arising from overlapping or translated records. Second, the English-only inclusion criterion may introduce language bias by excluding non-English publications. This limited data scope may inadequately represent global research on phage-microbiome regulation. Lastly, the bibliometric visualization software used in this study does not distinguish authorship positions, such as first or corresponding authors, but ranks authors collectively based on publication output and co-authorship relationships. As a result, the authors identified as prolific in this study reflect overall research productivity rather than specific authorship roles or individual research influence.

## 5 Conclusion

This review systematically examines current research, key focus areas, and future directions in phage-mediated gut microbiota regulation. As key microbiome regulators, phages uniquely maintain microbial homeostasis, modulate immunity, and combat antibiotic resistance. Global research output has increased steadily, led by China and the United States. Key research areas include: innovations in viroomics technology, the evolution of antibiotic resistance and microbiota-immune interactions, the expansion from phage therapy to microbiome interventions, and novel paradigms in immune regulation. Challenges remain, including geographic disparities, limited collaboration, incomplete mechanistic insights, and translational barriers. Future priorities include: enhanced multi-omics integration, interdisciplinary collaboration, standardization, and clinical translation of engineered phages. These advances will enable novel disease interventions and microbiome-based therapies.

## Data Availability

Publicly available datasets were analyzed in this study. This data can be found here: The original contributions presented in this study are included in this article/supplementary material, further inquiries can be directed to the corresponding author.
